# Outcomes of Bariatric Surgery in Patients with Schizophrenia

**DOI:** 10.3390/nu16152487

**Published:** 2024-07-31

**Authors:** Inka Miñambres, Miguel Ángel Rubio-Herrera, Joana Nicolau, Camila Milad, Maria José Morales, Marta Bueno, Alfonso Calañas, Mar Carceller-Sindreu, Ana de Hollanda

**Affiliations:** 1 Servicio de Endocrinología, Hospital de La Santa Creu i Sant Pau, 08041 Barcelona, Spain; 2 CIBER de Diabetes y Enfermedades Metabólicas (CIBERDEM), 28029 Madrid, Spain; 3 Departament de Medicina, Universitat Autònoma de Barcelona (UAB), 08193 Bellaterra, Spain; 4 Servicio de Endocrinología y Nutrición, Hospital Clínico San Carlos, 28040 Madrid, Spain; marubioh@gmail.com; 5 Departamento de Medicina, Universidad Complutense, 28040 Madrid, Spain; 6 Servicio de Endocrinología, Hospital de Son Llàtzer, 07198 Mallorca, Spain; jnicolauramis@gmail.com; 7 Servicio de Endocrinología, Hospital Clínic de Barcelona, 08036 Barcelona, Spain; milad@clinic.cat (C.M.); anidehollanda@gmail.com (A.d.H.); 8 Servicio de Endocrinología, Complexo Hospitalario Universitario de Vigo, 36312 Pontevedra, Spain; maria.jose.morales.gorria@sergas.es; 9 Servicio de Endocrinología y Nutrición, Hospital Universitari Arnau de Vilanova, 25198 Lleida, Spain; mbuenodiez@gmail.com; 10 Obesity, Diabetes and Metabolism (ODIM) Research Group, Institut de Recerca Biomèdica Lleida (IRB-Lleida), 25198 Lleida, Spain; 11 Servicio de Endocrinología, Hospital Universitario Reina Sofía, 14004 Córdoba, Spain; contentine@yahoo.es; 12 Servicio de Psiquiatría, Hospital de La Santa Creu i Sant Pau, 08041 Barcelona, Spain; mcarceller@santpau.cat; 13 Centro de Investigación Biomédica en Red de Salud Mental (CIBERSAM), 28029 Madrid, Spain; 14 Institut d’Investigació Biomèdica Sant Pau (IIB-Sant Pau), 08041 Barcelona, Spain; 15 Centro de Investigación Biomédica en Red Fisiopatología de la Obesidad y Nutrición (CIBEROBN), 28029 Madrid, Spain

**Keywords:** obesity, obesity surgery, schizophrenia, severe mental illness, weight loss

## Abstract

Background: Outcomes of bariatric surgery (BS) in patients with schizophrenia are poorly understood. We aimed to analyze the effects of BS in patients with schizophrenia (SZ) or schizoaffective disorder (SZA). Methods: This was a multicenter, retrospective case-control study in patients with SZ or SZA who had undergone BS in seven public referral hospitals in Spain. Controls without psychiatric comorbidity were selected in a 1:4 ratio. Detailed clinical and biochemical data were collected preoperatively and at 12, 24, 36, 48, and 60 months after BS. Results: Twenty patients with SZ (*n* = 15; 75%) or SZA (*n* = 5; 25%) and 80 matched controls were studied. There were no differences between patients and controls concerning the evolution of the percentage of total weight loss. The remission rate of the main comorbidities was similar between groups except for hypertension, which was lower in patients with a psychotic disorder from year 3. There were no mortalities within 30 days of surgery in either group. The psychiatric medication burden did not change during follow-up. Conclusions: BS is safe and effective in carefully selected patients with SZ. The course of the psychiatric disease does not seem to be worsened by the procedure.

## 1. Introduction

Obesity is a complex disease that poses a significant health and well-being risk. It is associated with many comorbidities, such as type 2 diabetes, dyslipidemia, hypertension, obstructive sleep apnea (OSA), cardiovascular disease, and increased mortality [1,2,3]. Many psychological comorbidities are associated with obesity, including eating disorders, substance abuse, depression, anxiety disorders, and severe mental illnesses such as bipolar disorder or schizophrenia (SZ) and schizoaffective (SZA) disorders [4,5,6].

The prevalence of SZ in the general population is 0.32% [7], and its association with obesity is bidirectional and is due to multiple causes, including the impact of antipsychotic medications and changes in lifestyle habits, with decreased physical activity and increased preference for certain hypercaloric foods often seen in patients with SZ [8,9,10,11]. Genetics or the altered hypothalamic–pituitary axis and the low-grade inflammatory state present in obesity can, in turn, impact the mental health of individuals living with obesity [8,9,10]. Moreover, obesity has a deleterious impact on patients with SZ by increasing their cardiovascular risk and even antipsychotic medication non-adherence [12,13,14].

Bariatric surgery (BS) is the most effective and longstanding treatment for severe obesity, with reduced morbidity and mortality and improved health-related quality of life [15,16,17,18]. However, the outcomes of BS in patients with SZ are poorly understood and are the focus of significant debate. A survey conducted on mental health professionals who perform preoperative evaluations of candidates for bariatric surgery denoted that 30.9% would consider the presence of a psychotic disorder as a clear contraindication to surgery [19].

Our primary objectives for this study were as follows: (1) to compare the effects of BS on weight loss and obesity-related comorbidities between patients with SZ or schizoaffective disorder (SZA) matched with patients without psychiatric comorbidity, (2) to compare postsurgical complications between both groups and (3) to describe the effects of BS on the course of the mental disorder, up to five years after surgery.

## 2. Materials and Methods

### 2.1. Study Design

We conducted a multicenter, retrospective case-control study, performed by the Obesity Area of the Spanish Society of Endocrinology and Nutrition (ObesitySEEN), with a review of cases with SZ or SZA who had undergone BS from 2007 to 2022 in seven public referral hospitals in Spain. Patients were included in the study if they had a diagnosis of SZ or SZA made by a psychiatrist before the performance of the BS and if they were followed up a minimum of 12 months after the BS. Controls were selected in a 1:4 ratio and included participants without psychiatric comorbidity matched with patients by the hospital of origin, age (±5 years), sex, type of surgery, and initial BMI (±5 kg/m^2^). Screening for psychopathology in patients and controls was made by the psychiatrist of each center and according to each center’s protocol. In all centers, the presence of active bulimia, binge eating, or other eating disorders, as well as substance abuse disorders, were considered contraindications for surgery. In the case of patients with SZ or SZA, the psychiatrist had to state that SZ or SZA was stable. Detailed clinical and biochemical data were retrospectively collected by reviewing the medical records. The study was conducted according to the principles of the Declaration of Helsinki, and all the patients signed an informed consent form approved by the institutional ethics committee. It was registered at clinicaltrials.gov NCT06043206.

### 2.2. Main Outcomes and Measures

We developed a questionnaire to collect the demographic data and anthropometric characteristics of the patients (weight, height, and body mass index (BMI)). We calculated the percentage of total weight loss (%TWL) as 100 × [(body weight at baseline − body weight at year 1 to 5)/body weight at baseline]. We calculated the percentage of excess weight loss (%EWL) by taking the ideal body weight equivalent to a BMI of 25 kg/m^2^. Clinical data concerning the metabolic comorbidities of obesity (type 2 diabetes (T2DM), hypertension, dyslipidemia, and obstructive sleep apnea syndrome (OSAS)) were also collected. We defined remission of comorbidities as cessation of medication for hypertension or hyperlipidemia with normal blood pressure and lipid levels, cessation for continuous positive pressure ventilation for sleep apnea, and normalization of glycosylated hemoglobin (HbA1c) (<6.5%) with no medications for diabetes. Laboratory tests were registered, including glycemia, HbA1c, and lipid profile. Data concerning SZ included its subtype (SZ or SZA), years from diagnosis, documented hospitalizations, and treatment. We calculated the psychiatric medication burden by generating a continuous variable using a previously validated formula [20]. In this formula, the daily dose of each drug is divided by the “recommended dose”, and each fraction result is summed to obtain a final number. It is one of the most correct approaches to analyze the medication load in patients who take drugs from different families (antipsychotics, antidepressants, benzodiazepines…). In this formula, the weight of each drug is considered separately and compared to the recommended daily doses, and their sum obtains a final total value that is a quantitative variable. From this variable, we can approximate the severity of the disorder or its evolution since higher medication loads reflect more drugs and more dosages necessary for the patient to maintain their stability.

Moreover, surgical and psychiatric complications after surgery were recorded and classified as those during hospitalization, <30 days after surgery, and during the rest of the follow-up. All data were collected preoperatively and at 12, 24, 36, 48, and 60 months after BS.

### 2.3. Statistical Analysis

We expressed variables as mean ± SD for continuous variables and absolute frequencies and percentages for categorical variables. We evaluated the normality of data distribution using the Kolmogorov–Smirnov test. We assessed the relationship between qualitative variables using the chi-squared test. The *t*-test and Mann–Whitney test were used to analyze independent samples. Paired *t*-test and Wilcoxon test were used to analyze changes between baseline and subsequent data at 12, 24, 36, 48, and 60 months. *p* Values < 0.05 were considered statistically significant. Data were analyzed using the Statistical Package for the Social Sciences (SPSS Inc., Chicago, IL, USA) version 25.0 for Windows.

## 3. Results

### 3.1. Characteristics of Participants

Twenty patients with SZ (*n* = 15; 75%) or SZA (*n* = 5; 25%) and 80 matched controls were studied. Patients had a mean duration of the psychiatric disorder of 12.17 ± 7.56 years and mean previous hospitalizations of 0.84 ± 1.26. Eleven patients (55%) had no previous hospitalizations due to their psychiatric disorder. All patients were considered eligible for surgery considering the combined evaluation of the usual psychiatrist of the patient and the psychiatrist adjunct to the obesity unit. Surgeries performed were 55% sleeve gastrectomy and 45% gastric bypass. Follow-up rates of the whole study group at 12, 24, 36, 48, and 60 months were 100%, 88%, 83%, 67% and 68%, respectively. Table 1 shows the baseline characteristics of patients and controls.

### 3.2. Weight and Comorbidities Evolution

**Figure 1:**
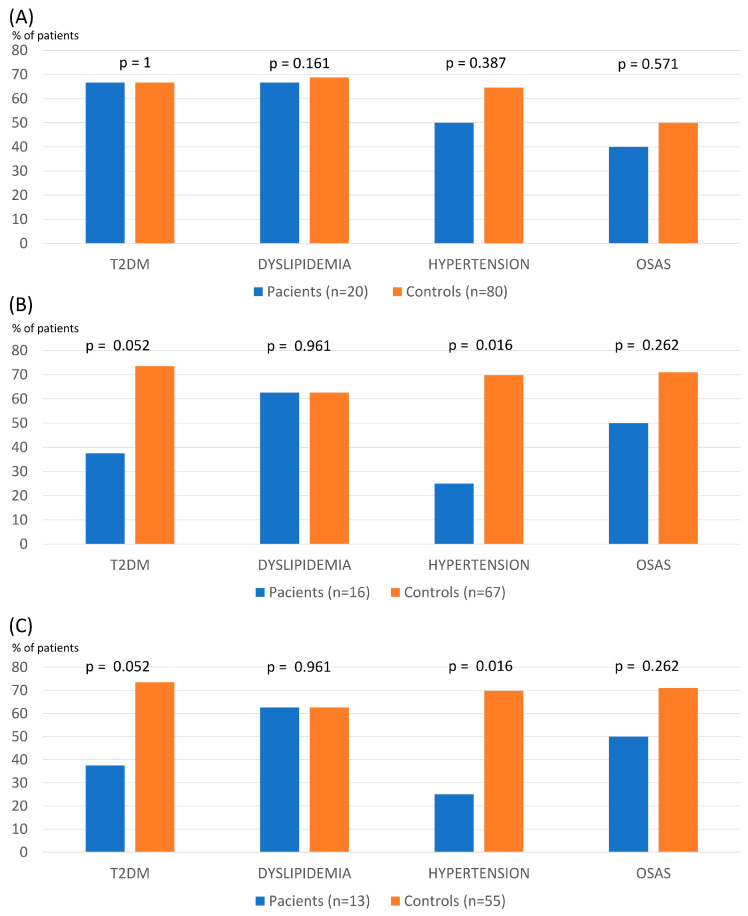
Remission of main obesity comorbidities at 1 (**A**), 3 (**B**), and 5 years (**C**) after bariatric surgery in patients with schizophrenia and controls (T2DM: type 2 diabetes; OSAS: obstructive sleep apnea syndrome).

There were no differences between patients with SZ/SZA and controls concerning the evolution of BMI, % TWL, or % EWL over 60 months (Table 2). The % TWL in patients with SZ/SZA and controls was 32.53 ± 7.74 vs. 31.29 ± 8.75 and 29.13 ± 10.51 vs. 27.07 ± 15.00 at 1 and 5 years after surgery, respectively. The remission rate of main comorbidities was similar between groups except for hypertension, which was lower in patients with a psychotic disorder from year 3 (Figure 1).

### 3.3. Surgical Complications

There were no mortality cases within 30 days of surgery in either group. Early postoperative complications were uncommon in both groups (patients: one anastomotic leak and one wound infection; controls: one anastomotic leak and one dysphagia). Late postsurgical complications included one gastric fistula, one patient with malnourishment in the SZ/SZA group, as well as one anastomotic ulcer, one patient with gastroesophageal reflux disease, and two with eventrations in the control group.

### 3.4. Other Follow-Up Data

Adherence to scheduled medical visits was 92.01 ± 17.53% in the SZ/SZA group vs. 86.38 ± 20.03% in the controls (*p* = 0.059). Adherence to postsurgical scheduled visits with the dietician was similar, 80.00 ± 25.82% in the SZ/SZA group vs. 79.09 ± 31.87% in the controls (*p* = 0.895).

**Figure 2:**
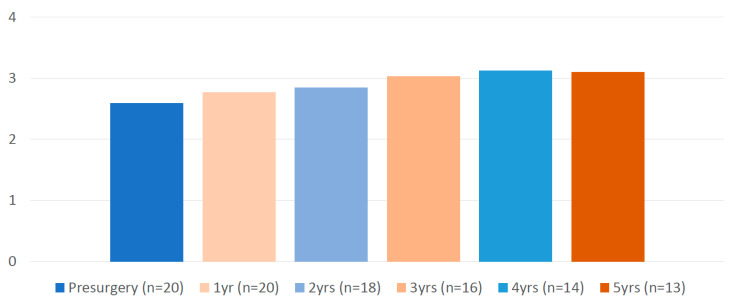
Evolution of the medication load of patients with schizophrenia before and after bariatric surgery (formula at Hanlon et al. 2009 [20]).

Concerning the evolution of psychiatric disease, two patients presented relapse of psychiatric symptoms during hospitalization (in the context of removal of psychiatric medication during the early postsurgical period). One patient relapsed during the early postoperative period (in the context of grief), and four patients presented psychiatric worsening that needed hospitalization during the five years of follow-up. As shown in Figure 2, the psychiatric medication burden did not change during follow-up.

## 4. Discussion

In this study of patients with co-morbid SZ or SZA undergoing BS, we show that BS is similarly safe and effective when compared with controls without psychiatric comorbidity. The course of psychiatric disease does not seem to be worsened by the surgical procedure. However, clinicians should pay careful attention to the management of antipsychotic medications in the early post-surgical period.

Currently, limited empirical data exist regarding the efficacy of BS in patients with SZ. In most cases, outcomes of patients with SZ have been analyzed in observational studies together with patients with bipolar disorders, finding they have a good prognosis after BS in terms of weight loss [21,22,23,24,25]. SZ and bipolar disorder (BD) are serious mental illnesses that share some characteristics, such as poor adherence to treatment, decreased quality of life, and life expectancy. However, they also present many differences we should not ignore when evaluating them as a single group regarding the response to BS. These are two very different pathologies that affect different brain circuits (affecting more predominantly the orbitofrontal cortex in bipolar disorder and the dorsal and frontotemporal areas in SZ) [26,27] and show very diverse symptoms [28]. Furthermore, patients with SZ have worse cognitive performance than patients with bipolar disorder from the preclinical phases and in their evolution [29]. The prognoses of these two diseases are very different, with a chronic course and poor prognosis being the rule in SZ (79% of patients), while 74% of patients with BD present a good or intermediate evolution [30,31].

Studies analyzing patients with SZ separately from other psychiatric disorders are scarce and limited to less than ten patients with a short follow-up. Two previous control-matched studies have analyzed the outcomes of patients with SZ compared with controls with no psychiatric disease. Archid et al. [32] reported outcomes of seven patients followed up to 24 months after surgery and found similar weight loss compared to controls and no exacerbation of psychiatric symptoms. Similarly, Hamoi et al. [33] found no differences in weight loss at 12 months after BS in their series of five patients with SZ. Other previous reports are limited to smaller case series and support the efficacy of BS in this population [34,35]. Therefore, we provided the most extensive series of subjects with SZ submitted to BS. We confirmed that weight loss was similar to patients without psychiatric comorbidity and that the effects on weight loss could extend up to five years of follow-up. Furthermore, we found similar resolution rates of main comorbidities, except for a lower remission of hypertension and a non-significant trend towards a lower remission of diabetes. Despite similar rates of weight loss, possible explanations for this finding may be that psychiatric patients continue on antipsychotic medications after BS, with their well-known effects on the metabolic syndrome features [36,37]. Another possible explanation may be that patients with SZ had a larger duration of metabolic comorbidities than controls, although this data could not be collected for the present study.

Postsurgical complications need to be assessed to account for the risk–benefit profile of BS in a specific population. Data from electronic health records from several US healthcare systems showed that patients with preoperative severe depression or anxiety or BD, psychosis, or SZ spectrum disorders had higher follow-up levels of emergency department visits and hospital days compared to those with no mental illness after BS procedures [24]. However, the emergency visits’ causes were not accounted for, and patients with different psychopathologies were analyzed together. Our study found that early and late postsurgical complications were balanced between patients with SZ and controls.

There is concern that BS and the significant alterations in eating patterns essential for a successful postoperative course could be major stressors for patients with SZ and might increase the risk of relapse in the immediate postoperative period. In this regard, Brito et al. [34], in their observational study of five patients with SZ, found no relapse of psychiatric symptoms when assessed using the Positive and Negative Syndrome Scale (PANSS). Neither did Archid et al. [32] find an exacerbation of psychiatric symptoms in the postsurgical period. However, in the non-controlled study of Shelby et al. [24], the three SZ patients reported experiencing postsurgical exacerbation of psychiatric symptoms, and one patient required psychiatric hospitalization. In our study, one patient presented a relapse of psychiatric symptoms during hospitalization related to withdrawal of his usual treatment in the immediate postsurgical period. One patient relapsed during the early postoperative period in the context of mourning. There is concern that the management of antipsychotic medications in the immediate postsurgical period may account for early postoperative decompensations. Therefore, although no specific guidelines exist, the general recommendation would be to maintain antipsychotic medications until 24 h before surgery, resume them as early as possible, and perform close monitoring of blood levels of drugs to control the possible effect of altered drug absorption after surgery [33]. In case of prolonged oral intolerance, consultation with the psychiatry service is advisable to find alternatives to the oral route. About the four patients who worsened during the five years of follow-up, we believe that this was a normal evolution of these disorders, characterized by relapses, and that it was not related to the surgery itself [38,39].

Given the bidirectional association between obesity and SZ [8,9,10], and the possible impact of obesity on the prognosis of psychiatric disease, one might expect that, in the long term, weight loss might lead to improvement in psychiatric symptoms. Archid et al. [32] found a significant improvement in the self-estimated mood and satisfaction observed in all seven study participants. Brito et al. [34] found no significant changes in antipsychotic drugs during follow-up. In our study, the medication burden did not increase during the five years of follow-up, indicating at least the stabilization of SZ. In fact, we might even expect an improvement in these patients. A recent systematic review found a relationship between a worse metabolic profile and worse cognitive performance in patients with SZ [40]. Based on this relationship, an improvement in this performance, including social functioning, would be expected in patients with SZ who improved their metabolic profile after CB. However, one of our study limitations is the lack of specific scales to measure psychotic and depressive symptomatology. That is why we used a robust variable such as medication load to approximate the evolution of the psychiatric illness after the intervention, as has been usually used in other articles in various disciplines [20,41,42,43].

The evaluation of patients with severe mental illnesses such as SZ and their suitability for BS is a matter of debate. Patients with SZ have declined functionality and more difficulty maintaining healthy lifestyle habits compared to patients without psychological comorbidities. This fact, the disease’s poor prognosis in terms of the chronicity of the symptoms, and the patient’s social and work adaptations may be the main reasons BS is automatically denied in some centers [19]. However, the decreased functionality of patients with SZ is not directly related to compliance with post-surgery indications. Therefore, these patients should not be ruled out for fear of obtaining poor post-surgery results. Guidelines support that any patient considered for a bariatric procedure with a known or suspected psychiatric illness should undergo a formal mental health evaluation before the procedure [44,45]. In our experience, patients with SZ follow simple guidelines and present results equivalent to healthy controls regarding weight loss and even adherence to medical follow-up. All the patients in our series were considered eligible for surgery considering the combined evaluation of the patient’s usual psychiatrist and the psychiatrist adjunct to the obesity unit. It is of particular importance that the mental health provider that assesses the eligibility for BS is familiar with BS behavioral health to determine the candidate’s ability to cope with the adversity of surgery, changing body image, and required lifestyle changes [45].

The present study’s limitations include its observational nature and sample size. However, we provide the most extensive series of patients with SZ or SZA submitted to BS. Main strengths include the long-term follow-up and the presence of a control group matched by age, sex, type of surgery, and baseline BMI.

## 5. Conclusions

BS in carefully selected patients with SZ or SZA is a safe and effective treatment for obesity. We demonstrated long-term weight loss, meaningful improvement in obesity-related comorbidities with no deleterious effects on psychiatric symptoms, and low rates of surgical complications. Results are comparable to patients without psychopathology except for a lower remission rate of hypertension observed since the third year post-surgery. Therefore, stable SZ may no longer be seen as an absolute contraindication of BS since the careful presurgical selection of patients can lead to successful clinical outcomes. Future prospective studies with uniform criteria for evaluating and monitoring patients with SZ will help confirm our findings.

## Figures and Tables

**Table 1:** Baseline characteristics of patients with schizophrenia or schizoaffective disorder and matched controls.

	SZ-Group (*n* = 20)	Control Group (*n* = 80)	*p* Value
Age (years)	48.7 ± 7.0	49 ± 7.11	0.866
Sex (*n* (%) females)	14 (70)	58 (72.5)	0.824
BMI (kg/m^2^)	46.68 ± 5.18	46.07 ± 5.23	0.644
Type of surgery (*n* (%))	11 (55) sleeve 9 (45) bypass	44 (55) sleeve 36 (45) bypass	1
Hypertension (%)	10 (50)	48 (60)	0.418
T2DM (*n* (%))	9 (45)	36 (45)	1
Dyslipidemia (*n* (%))	9 (45)	48 (60)	0.266
OSAS (*n* (%))	10 (50)	40 (50)	1
FPG (mg/dL)	112.73 ± 27.44	115.41 ± 34.70	0.833
HbA1c (%)	5.99 ± 0.74	6.07 ± 0.91	0.977
TC (mg/dL)	182.22 ± 36.42	200.16 ± 37.00	0.079
Triglycerides (mg/dL)	121.92 ± 56.51	153.38 ± 70.78	0.049
LDLc (mg/dL)	110.52 ± 22.28	119.45 ± 28.66	0.210
HDLc (mg/dL)	47.45 ± 10.86	47.26 ± 12.95	0.952

BMI: body mass index; T2DM: type 2 diabetes; OSAS: Obstructive sleep apnea syndrome; FPG: fasting plasma glucose; TC: total cholesterol, LDLc: low-density lipoprotein cholesterol; HDLc. High-density lipoprotein cholesterol.

**Table 2:** Evolution of body mass index, % total weight loss, and % excess weight loss of patients and controls during the 5 years of follow-up.

	Baseline		1 Year		2 Years		3 Years		4 Years		5 Years	
	Patients *n* = 20	Controls *n* = 80	Patients *n* = 20	Controls *n* = 80	Patients *n* = 18	Controls *n* = 70	Patients *n* = 16	Controls *n* = 67	Patients *n* = 14	Controls *n* = 53	Patients *n* = 13	Controls *n* = 55
BMI	46.68 ± 5.18	46.07 ± 5.23	31.42 ± 5.51	31.56 ± 5.30	32.10 ± 5.41	31.21 ± 5.21	32.80 ± 7.17	32.15 ± 5.71	33.15 ± 6.87	32.65 ± 5.46	33.59 ± 6.99	33.93 ± 6.35
% TWL			32.53 ± 7.74	31.29 ± 8.75	30.78 ± 7.51	31.32 ± 10.24	32.13 ± 10.63	28.77 ± 10.13	29.73 ± 10.47	27.60 ± 9.40	29.13 ± 10.51	27.07 ± 15.00
% EWL			73.53 ± 22.27	70.93 ± 20.28	70.16 ± 22.80	71.17 ± 23.77	74.06 ± 32.88	66.37 ± 24.28	66.51 ± 26.47	64.04 ± 3.37	64.93 ± 26.36	61.80 ± 33.10

BMI: body mass index; %TWL: % total weight loss; %EWL: % excess weight loss. All *p* comparing patients and controls at each time point were >0.05.

## Data Availability

The raw data supporting the conclusions of this article will be made available by the authors, without undue reservation.
